# New aneurysm formation after endovascular embolization of a vertebral epidural AV fistula: a rare sequelae of NF AV fistulae

**DOI:** 10.3389/fneur.2023.1132334

**Published:** 2023-06-07

**Authors:** Yiyong Zeng, Xianru Li, Junjun Zhang, Yi Huang, Zhiqin Lin, Shengjun Zhou

**Affiliations:** ^1^Department of Neurosurgery, The First Affiliated Hospital of Ningbo University, Ningbo, China; ^2^Key Laboratory of Precision Medicine for Atherosclerotic Diseases of Zhejiang Province, Ningbo, China

**Keywords:** neurofibromatosis type 1 (NF1), vertebral arteriovenous fistulae, endovascular treatment, subarachnoid hemorrhage, aneurysm

## Abstract

**Background:**

Neurofibromatosis type 1 (NF-1) is a dominant genetic disorder often accompanied by lesions of the neurovascular system. Patients with NF-1 are predisposed to unique vertebral artery fistula (AVF).

**Case description:**

We report on a rare case of multiple neurovascular abnormalities in a 47-year-old man with neurofibromatosis. He was admitted due to a sudden headache and was found to have suffered a subarachnoid hemorrhage from a left vertebral arteriovenous fistula. He underwent two endovascular procedures complicated by a delayed extraspinal mass 7 days after treatment. Angiography revealed a new vascular abnormality, and although we performed another embolization, it failed to respond to further embolization.

**Conclusion:**

Vascular abnormalities in patients with NF-1 can be complex. Endovascular intervention remains feasible for NF-1 related AVF, however, partial occlusion of the fistula should be avoided to limit and iatrogenic damage to the blood vessels.

## Introduction

Neurofibromatosis type 1 (NF-1) is an autosomal dominant genetic disorder with widespread neuroectodermal and mesodermal dysplasia affecting the skin, nervous system, bone, and vascular system (1). Patients with NF-1 are predisposed to unique spinal epidural arteriovenous malformations (DAVF) (2). Vertebral arteriovenous fistulas (AVF) are rare type of epidural arteriovenous malformations usually located in the spine and widely accepted to require treatment (3). It is mainly caused by direct abnormal communication between branches of vertebral artery and spinal radiculomeningeal vein or epidural veins (3).

We discuss the clinical presentation and challenges of endovascular management of AVF in a case multiple NF-1 associated vascular lesions.

## Case presentation

The patient was a 47-year-old man who was admitted to hospital following to a sudden headache 3 days prior. There was a previous history of resection of a tumor from the left side of the patient’s neck 20-years before the current presentation.

**Figure 1:**
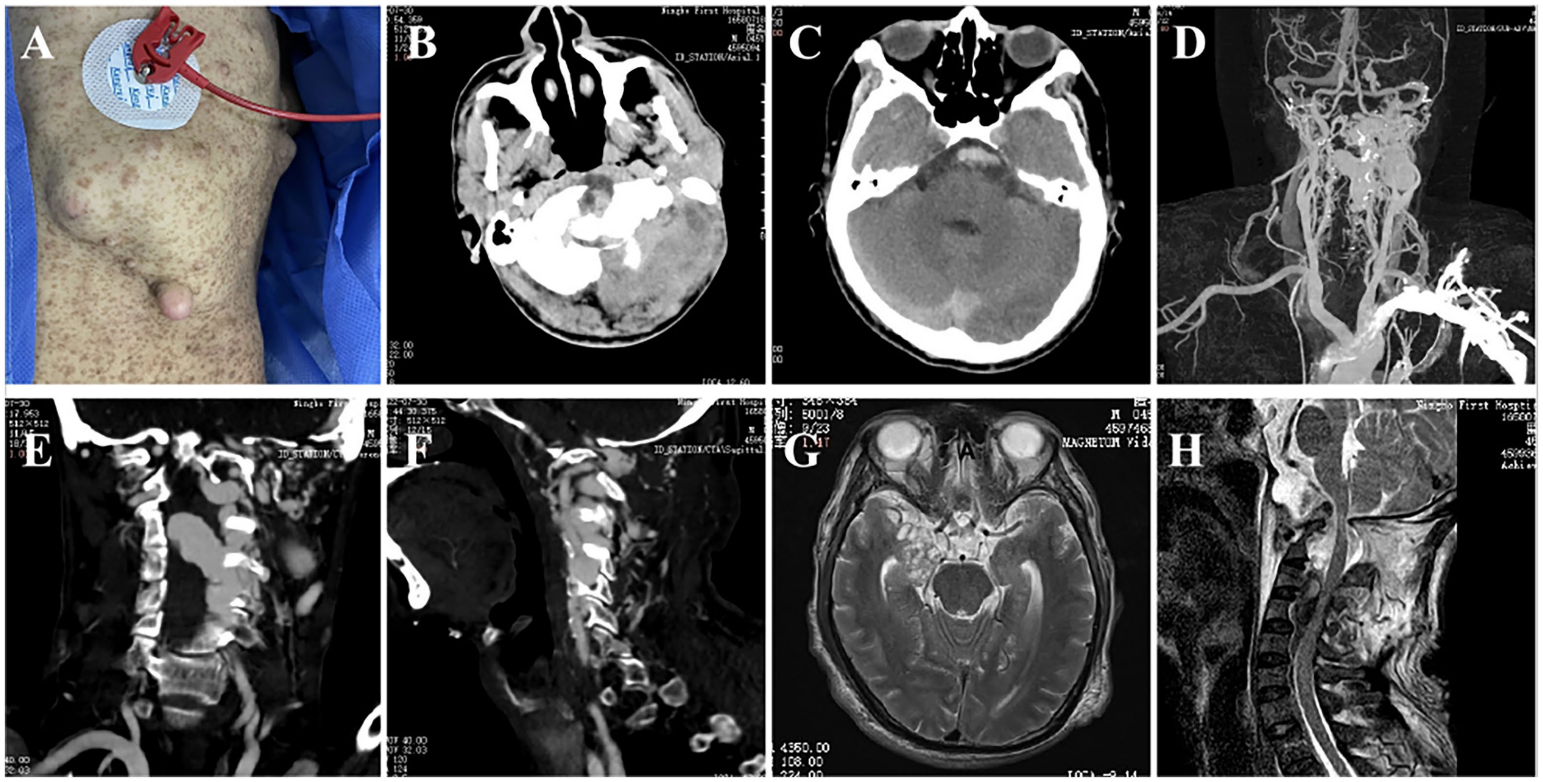
The patient’s body surface features and imaging findings. **(A)** Multiple neurofibromas and typical café-au-lait spots; **(B)** Left skull bone destruction and occipital muscle swelling; **(C)** CTA showed subarachnoid hemorrhage; **(D–F)** CTA showed multiple vascular abnormalities in the left skull base and neck, and the thick venous lake compressed the spinal cord; **(G)** MRI showed the presence of an intracranial tumor; **(H)** Atlantoaxial dislocation, fluid void signal in the spinal canal and compression of the spinal cord.

Clinical examination revealed multiple neurofibromas and typical café-au-lait spots on the patient’s skin (Figure 1A). Similar spots were also present on the skin of his immediate family members. The muscle strength of his limbs showed a progressive decline. On admission, only the left upper limb was affected, and the muscle strength was grade 1. By the third day, the muscle strength of both the left lower limb and the right upper limb had decreased to grade 1. He was otherwise fully conscious (GCS 15) with no other neurological deficits.

**Figure 2:**
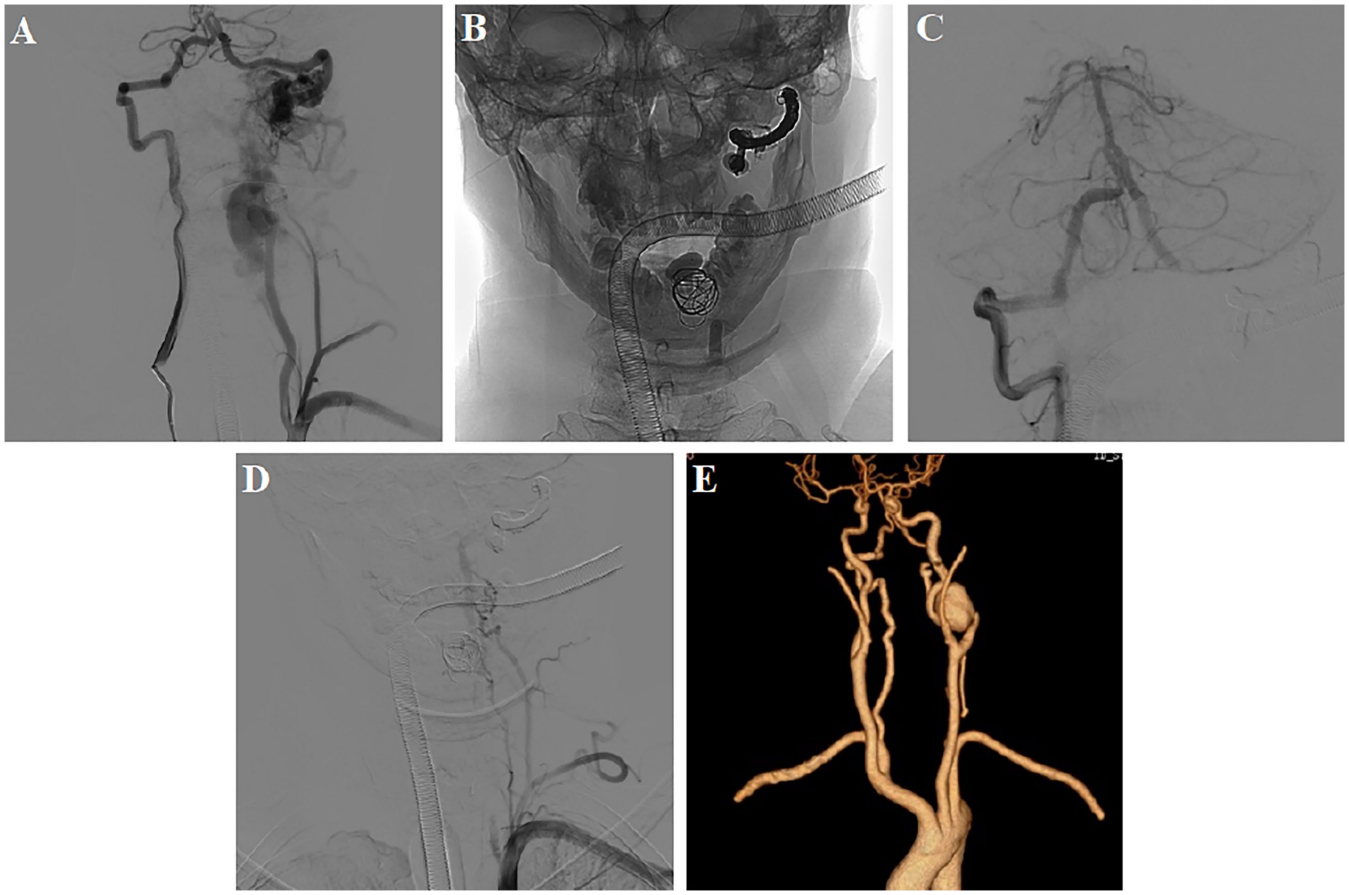
First postoperative DSA and CTA examination. **(A)** DSA demonstrated multiple high-flow arteriovenous fistulae at the left CVJ (fistula A) and the C3–C4 level (fistula B); **(B–D)** Balloon combined with a coil and Onyx embolization, leaving an anastomotic muscular branch backflow vertebral artery supplying blood to fistula B; **(E)** CTA showed the disappearance of abnormal blood vessels in the left neck.

Computed tomography (CT) head showed subarachnoid hemorrhage (modified Fisher grade 2). The magnetic resonance imaging revealed space-occupying lesions deep in the right temporal lobe, flow voids in the craniovertebral junction (CVJ), and the left spinal canals from C3 to C4 (Figures 1G,H). CT angiography revealing a left cervical dural arteriovenous fistula, an aneurysm in the left internal carotid artery, multiple bone abnormalities in head and neck (Figures 1B–F). Digital subtraction angiography demonstrated the presence of multiple high-flow arteriovenous fistulae at the left CVJ (fistula A) and the C3–C4 level (fistula B), a direct shunt between the left vertebral artery and the epidural vein, forming a huge venous lake connected to the paravertebral venous plexus on the left (Figure 2A); the right vertebral arteriography exhibited a leftward retrograde flow of blood.

The patient was diagnosed with NF-1 accompanied by left AVF and subarachnoid hemorrhage. The compression of the spinal cord by the lacunae resulted in progressive quadriparesis, while the resulting high perimedullary venous pressures leading to the subarachnoid hemorrhage.

## Treatment

Intervention was recommended to prevent further hemorrhage and arrest myelopathy. The complex architecture, extensive number lesions was felt increase difficulty and morbidty associated with open surgery. Previous reports in the literature of successful endovascular treatment of such AVFs, underpinned our choice of embolization as a safe treatment option for this patient.

The first treatment was performed 4 days after admission. Three detachable balloons were placed at the proximal end of fistula A，the venous lake of fistula B，and origin of the vertebral artery. One coil was added to support and fix the balloon in venous lake. Then, fistula A was completely occluded via the right vertebral artery using coils and Onyx (Supplementary Video 1). Post-operative angiography revealed the complete occlusion of fistula A and the beginning of the left vertebral artery. An anastomotic muscular branch backflow vertebral artery continued to supply blood to fistula B (Figures 2B–D). At 7 days, the carotid artery CT angiography showed the disappearance of abnormal blood vessels in the left neck (Figure 2E).

**Figure 3:**
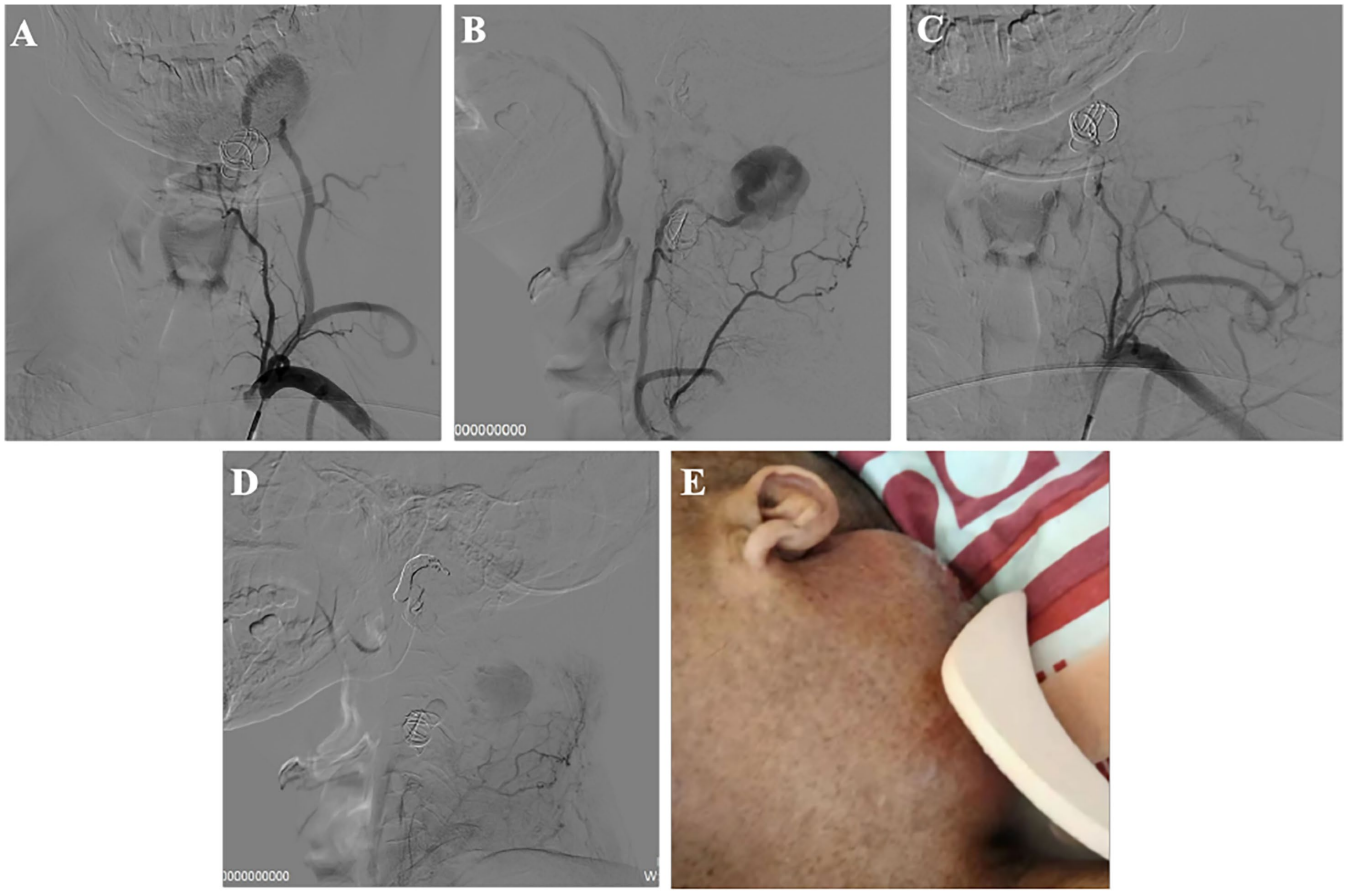
Recurrent vascular abnormality after the first operation and the second interventional treatment. **(A,B)** DSA showed muscular branch artery ruptured, forming a huge aneurysm; **(C,D)** After second treatment; **(E)** Left neck mass.

Eleven days post-procedure, a pulsating mass was observed at the left posterior auricular arear. Follow-up angiography revealed this to be a ruptured aneurysm from the distal muscular branch artery of the costocervical trunk (Supplementary Video 2). Further embolization of this aneurysm was performed using a gelatin sponge (Figures 3A–D).

## Outcome and follow-up

The patient had left hemiplegia before treatment and recovered to grade 3 after first treatment. The mass was present without pulsation after second treatment. At 14 days, the mass in the left neck was further enlarged (Figure 3E). The patient declined further intervention following this and was kept under clinical and radiological surveillance with no further increases in the mass noted.

## Discussion

Neurofibromatosis type 1, also termed von Recklinghausen’s disease, is a genetic disorder following an autosomal dominant pattern. It arises from NF-1 gene mutations at the long arm of chromosome 17 (17q11.2) that induces developmental abnormalities of the vascular system, such as arterial occlusion, intracranial vascular dysplasia, moyamoya vasculopathy, and aneurysms (4).

Patients with NF-1 are predisposed to distinctive vertebral arterial fistulae (5). Typical manifestations include radiculopathy, neck pain, and neck mass, while AVF rupture cases are characterized by neck hematoma, SAH, and hypotension (2). The pathogenesis of NF-1-related AVF has not been fully understood. It is thought that fragility and defects of the arterial wall related to NF-1 can cause AVF (6). Swain et al. (7) proposed two possible mechanisms by which an AVF might arise in patients with NF-1: abnormalities of connective tissue, including dysplastic smooth muscle in the arterial wall. Alternatively, the abnormal arteriovenous communications are primarily congenital in nature and arise directly as a manifestation of mesodermal dysplasia association with NF-1 among young patients.

The multiple arteriovenous fistulae in the present case support the congenital theory while and recurrent vascular abnormalities after intervention was in keeping with fragility of dysplatic vessels. The patient had two fistulas located at the craniocervical junction (fistula A) and the C3–C4 level (fistula B). Cerebral angiography showed retrograde venous drainage to the skull base, suggesting high venous pressure as the cause of the subarachnoid hemorrhage. The large lake of veins compresses the spinal cord, leading to progressive loss of strength in the limbs. The most effective treatment of AVF is to seal the two fistulas separately. The large size of the venous lake, the ongoing cord compression from this lacuna limited the choice of embolic devices to balloons and coils to avoid exacerbating the mass effect from the AVF. Unfortunately, the high flow in the fistula dislodged the balloon beyond the fistula into the venous lake. The fistula remained patent with ongoing supply from the muscular branches of the costocervical trunk artery (but slightly increased resistance at the venous side). Attempts at occluding this branch with a microcatheter were unsuccessful. The flow across the fistula was however reduced at the end of the procedure. Angiography showed that this muscular branch was not only supplying blood to the fistula, but also had many branches to the neck muscles. Magnetic resonance examination showed that the muscle signal in this part of the patient was abnormal increasing concerns that occlusion of this blood vessel would likely result in ischemic necrosis. Even if Onyx is injected from this blood vessel, it cannot diffuse to the fistula to achieve embolization effect.

Aneurysmal formation on this preserved muscle branch so shortly after surgery is hypothesized to be related to abnormal vascular development and postoperative hemodynamic changes in NF-1 patients. This complication highlights some of the challenges associated with managing this patient group and provides insights to guide future therapeutic endeavors.

## Conclusion

The vascular network of NF-1 related AVF is complex, and more may be prone to remodeling and *de novo* vascular lesions in patients with multiple fistulas. Endovascular embolization for NF-1 with AVF is feasible, but the fistula should be completely occluded during the treatment and any iatrogenic injury to the vessels.

## Data availability statement

The original contributions presented in the study are included in the article/Supplementary material; further inquiries can be directed to the corresponding authors.

## Ethics statement

The studies involving human participants were reviewed and approved by the Ethics Committee of Ningbo First Hospital. The patients/participants provided their written informed consent to participate in this study. Written informed consent was obtained from the individual(s) for the publication of any potentially identifiable images or data included in this article.

## Author contributions

ZL and SZ made substantial contributions to design and drafted the manuscript. YZ, XL, and JZ collected data. YH revised the manuscript critically for important intellectual content. All authors contributed to the article and approved the submitted version.

## Funding

This study was supported by the grants from the Ningbo Health Branding Subject Fund (PPXK2018-04), Ningbo Top Medical and Health Research Program (2022020304), Medicine and health science and technology projects of Zhejiang province (2019KY160), Ningbo Science and Technology Innovation 2025 Major Project (2022Z134), and Key Laboratory of Precision Medicine for Atherosclerotic Diseases of Zhejiang Province (2022E10026).

## Conflict of interest

The authors declare that the research was conducted in the absence of any commercial or financial relationships that could be construed as a potential conflict of interest.

## Publisher’s note

All claims expressed in this article are solely those of the authors and do not necessarily represent those of their affiliated organizations, or those of the publisher, the editors and the reviewers. Any product that may be evaluated in this article, or claim that may be made by its manufacturer, is not guaranteed or endorsed by the publisher.
